# Predictors of low antiretroviral adherence at an urban South African clinic: A mixed-methods study

**DOI:** 10.4102/sajhivmed.v23i1.1343

**Published:** 2022-02-10

**Authors:** Connor P. Bondarchuk, Nwabisa Mlandu, Tasneem Adams, Elma de Vries

**Affiliations:** 1 Department of Medicine, Harvard University, Boston, Massachusetts, United States of America; 2 Division of Neuropsychology, Faculty of Humanities, University of Cape Town, Cape Town, South Africa; 3 Cape Town Metro Health Service, Western Cape Department of Health, Cape Town, South Africa; 4 School of Public Health and Family Medicine, Faculty of Health Sciences, University of Cape Town, Cape Town, South Africa

**Keywords:** treatment adherence, antiretroviral therapy, mixed methods, HIV stigma, discrimination, health access

## Abstract

**Background:**

Low adherence to antiretroviral treatment (ART) in people living with HIV (PLHIV) remains a critical issue, especially in vulnerable populations. Although ART is responsible for greatly reducing the mortality and morbidity associated with HIV, low treatment adherence continues to impact the effectiveness of ART. Considering that a high level of adherence to ART is required for the excellent clinical outcomes with which ART is often associated, understanding the complex contextual and personal factors that limit high levels of treatment adherence remains paramount. Poor adherence remains an issue in many South African communities many years after the introduction of ART.

**Objectives:**

Our study sought to understand the specific factors and the interactions among them that contribute to non-adherence in this patient population in order to devise successful and contextually appropriate interventions to support ART adherence in PLHIV.

**Methods:**

This mixed-methods study employed a study-specific questionnaire (*N* = 103) and semi-structured interviews (*N* = 8) to investigate the factors linked to non-adherence at the Heideveld Community Day Centre in Cape Town, South Africa.

**Results:**

Over half (57.3%) of participants were ART non-adherent. Non-adherence was correlated with younger age, negative self-image and a low belief in the necessity of ART (*P* < 0.05). In patient interviews, alcohol use, treatment fatigue and stigmatisation emerged as contributors to suboptimal adherence.

**Conclusion:**

The results suggest that there remains a need for context-sensitive interventions to support PLHIV in South African communities. Future research needs to ensure that these targeted interventions take these factors into consideration.

## Introduction

Adherence to antiretroviral treatment (ART) in people living with HIV (PLHIV) promotes suppression of the HIV viral load and reduces patient morbidity and mortality. Yet despite ART’s promotion of greatly improved health outcomes for PLHIV, low treatment adherence undermines its effectiveness.

South Africa runs the largest public ART programme in the world, with around 62% of PLHIV having been initiated onto treatment.^1^ Nevertheless, although national and international initiatives have largely increased access to ART in countries like South Africa, treatment adherence remains a problem in resource-limited settings.^2,3,4^ Therefore, an updated and contextual understanding of the unique, highly personal barriers to treatment adherence is critical to the success of any potential intervention that seeks to reduce disengagement from ART.

When ART was first being implemented in sub-Saharan Africa, levels of self-reported adherence were generally shown to be high.^5,6^ Nevertheless, many years after the introduction of ART, poor adherence remains an issue in many resource-limited communities.^7,8,9^ Known predictors of low ART adherence include fear of discrimination, internalised stigma, personal attitudes about ART, clinic-related factors, structural factors and restricted access to treatment.^4,5,8^ Although the degree of stigma experienced by HIV-positive individuals varies significantly, stigmatisation and discrimination as a result of one’s HIV status have long been shown to negatively impact patients’ adherence to ART.^10^ Several clinic-related factors related to the healthcare system have also been reported to be associated with non-adherence in PLHIV. These include the unavailability of drugs, long queues, health worker attitudes towards HIV, limited clinic opening hours and lack of privacy.^4,11^

Importantly, structural barriers have been highlighted by the literature as potential factors contributing to difficulties with adherence. Poverty, transportation costs faced by low-income individuals and high levels of alcohol abuse are some of the factors previously shown to be associated with disengagement from ART.^3,4,12^ Therefore, a combination of individual-level factors, such as side effects, a lack of social support, local conditions, and structural and systemic barriers, likely interact to contribute to a patient’s overall level of adherence. Considering that clinic–patient discussions about personal barriers to adherence have been previously shown to mediate adherence levels,^5,13^ understanding the factors contributing to a patient’s low adherence and the interaction among those factors is crucial if successful and contextually appropriate interventions to support adherence are to be devised.

This mixed-methods study sought to expand and update knowledge about the contributors to poor adherence to ART in sub-Saharan Africa by investigating the factors currently mediating differential levels of adherence in a diverse urban patient population. Considering that the factors contributing to non-adherence may manifest differently based on temporal context and locale, it is crucial to understand the current factors contributing to suboptimal ART adherence so that health systems may plan efficacious interventions for their patient populations.

## Methods

This was a sequential, exploratory mixed-methods study that employed a combined quantitative and qualitative research design in distinct phases. The initial phase was a quantitative design and was questionnaire-based. The second phase was qualitative in nature and was conducted to expand on our quantitative data.

### Study setting and population

The study was conducted at the Heideveld Community Day Centre (CDC), a public 8-h day clinic operating in the Cape Town Metro Health District in Cape Town, South Africa. Located in the community of Heideveld, the clinic acts as a first line of access for PLHIV in Heideveld and in surrounding townships. The clinic serves a mostly low-income population and offers various health services, including chronic care, emergency care, ART services and social work counselling.

Patients who are HIV positive attend clinic appointments with registered nurses or a medical doctor. Antiretroviral treatment is also distributed to patients on site.

### Eligibility

A convenience sample was used for both phases. Initial identification of potential participants and recruitment procedures occurred outside the consultation room after patients’ appointments at the antiretroviral (ARV) clinic. Participants in the quantitative phase met the following eligibility criteria: age ≥ 18 years old and previously diagnosed with HIV. All participants were registered as patients at the Heideveld CDC.

Qualitative data collection was limited to HIV-positive individuals who met the eligibility criteria above and indicated non-adherence to their ART based on their responses in the preceding quantitative phase. Non-adherence was assessed based on three adherence questions:

- Sometimes if you feel worse, do you stop taking your HIV medication? (yes/no)
- Over the past weekend, did you take all of your HIV medication? (yes/no)
- In the last 30 days, on how many days did you miss at least one dose of any of your ARV medication? Write in number of days: ____

Non-adherents were classified as those who answered ‘yes’ to question 1 and/or ‘no’ to question 2 and/or reported missing two or more doses in the preceding 30 days.

### Recruitment and data collection

#### Quantitative

Quantitative data, including demographic information, stigma assessments, and patients’ beliefs and concerns about their ART, were collected between September and December 2019 at the Heideveld CDC. Participants were recruited by research staff operating inside the clinic within discreet and private rooms. Research staff utilised information sheets in the patient’s language of choice (English, Afrikaans or isiXhosa) to describe the study’s goals and the option for participation in the study. Interested patients were directed to appropriate members of the team, who provided additional information about the exact nature of the study.

Informed consent procedures as well as questionnaires were administered to participants by trained research staff members conversant in English, Afrikaans and/or isiXhosa. All processes took place in private rooms within the clinic, and participants were advised that they could withdraw at any point. After completing a questionnaire, participants were offered R80.00 (about $6.00) as compensation for their time.

#### Qualitative

Qualitative data collection was conducted between January 2020 and March 2020 in the Heideveld CDC. Individuals who self-reported as ART non-adherent based on the study criteria and who previously agreed to be contacted telephonically were eligible for participation in the qualitative phase. Semi-structured interviews (*n* = 8) lasted approximately 45 min and occurred in private office spaces or other locations within the clinic. Interviewees received R100.00 (around $7.00) for participation.

### Quantitative measures

All questionnaires were translated from English to Afrikaans and isiXhosa by professional translators. The surveys were then back-translated by other translators to confirm the accuracy of the translations.

#### Demographics

The questionnaire collected demographic data, including age, sex, education level, the number of months on ARVs and estimated income levels.

#### Adherence levels

Adherence to ARVs was assessed using three different questions (Table 1). The first two questions, which were answered dichotomously (yes/no), are based on assessments developed and used in other adherence studies.^14^ The final question, which asks patients to estimate how many days they have missed their ARV medication out of the past 30 days, has been validated against electronic drug monitoring as part of a three-item self-report measure.^15^ Adherence levels (adherent or non-adherent) were characterised based on answers to these three questions (Table 1).

**TABLE 1:** Participant characteristics.

Variable	*n*	%	Mean	Range	s.d.
**Age (years)**			42.7	18–66	10.6
Gender	-	-	-	-	-
Male	26	25.2	-	-	-
Female	77	74.8	-	-	-
**Language**					
English	40	38.8	-	-	-
Afrikaans	12	11.7	-	-	-
isiXhosa	51	49.5	-	-	-
**Highest level of education**					
No schooling	1	1.0	-	-	-
Some primary	7	6.8	-	-	-
Complete primary	9	8.7	-	-	-
Some secondary	36	35.0	-	-	-
Complete secondary	41	39.8	-	-	-
Higher	9	8.7	-	-	-
**Time since ARV initiation**					
0–6 months	14	13.6	-	-	-
6–12 months	10	9.7	-	-	-
12–18 months	7	6.8	-	-	-
18–24 months	5	4.9	-	-	-
≥ 2 years	67	65.0	-	-	-
**Income levels, annual**					
< R5000.00†	67	65.0	-	-	-
R5000.00 – R20 000.00	20	19.4	-	-	-
R20 000.00 – R35 000.00	9	8.7	-	-	-
R35 000.00 – R50 000.00	5	4.9	-	-	-
R50 000.00 – R65 000.00	1	1.0	-	-	-
> R65 000.00	1	1.0	-	-	-

*N* = 103

s.d., standard deviation; ARV, antiretroviral.

† , Incomes given in South African rands. At the time of data collection, R14.50 = US $1.

#### HIV-related stigma

The HIV stigma questionnaire consists of 12 items, with four groups of three items each comprising different subscales: personalised stigma, disclosure concerns, concerns with public attitudes and negative self-image. The 4-point Likert item set was previously adapted from the 40-item HIV Stigma Scale (HSS) and has been shown to have acceptable internal consistency (Cronbach’s α > 0.7) and comparable psychometric properties to the full-length scale. Differential item functioning for this abridged measure is negligible in different cultural contexts and in different languages.^16^ Lower subscale and sum scores reflect higher levels of stigma. Individuals with sum scores of ≤ 23 were classified as having high experienced stigma, whereas individuals with sum scores of ≥ 36 were classified as having low levels of stigma.

#### Accessibility factors

The accessibility questionnaire consists of six questions related to patients’ access to the clinic and their medications, as well as their ability to understand clinic staff. The accessibility questionnaire was created by the research team based on a literature review of barriers to treatment adherence in the South African context.

#### Behaviour and knowledge

The behaviour component of the questionnaire consists of 10 questions from the ‘Specific Concerns’ and ‘Specific Necessity’ components of the Beliefs about Medicine Questionnaire (BMQ-Concerns and BMQ-Necessity, respectively).^17^ This 5-point Likert item set has been shown to have high internal consistency in many contexts and high test–retest reliability.

The knowledge section was developed by the research team and consists of four true/false items testing patient knowledge about HIV and ARVs.

### Data analysis

#### Quantitative

Data were processed and analysed using IBM Corporation’s Statistical Package for the Social Sciences (SPSS) 24 for Mac. Descriptive frequencies were calculated for demographic characteristics, answers to dichotomous adherence questions and responses to 4-point and 5-point Likert items in the stigma, accessibility and behaviour portions of the questionnaire. The distribution of the summed scores on the HIV stigma survey portion and the BMQ-Necessity and BMQ-Concerns sections was determined to be roughly normal. Pearson’s coefficients and Spearman’s coefficients were calculated, and significance levels were set at *p* < 0.05.

#### Qualitative

Qualitative narrative data were generated using transcripts from semi-structured interviews. Two researchers double-coded eight transcripts independently and compared them side by side to identify inconsistencies. Codes were grouped thematically based on their viability in responding to the research question, and the relationships between codes were identified. Illustrative participant quotes are included below.

### Ethical considerations

The study received ethics approval from the Dartmouth College (#00031086) and University of Cape Town Institutional Review Boards (HREC 464/2018) and provincial approval from the Western Cape Department of Health.

## Results

### Quantitative findings

The 103 subjects in this study had a median age of 42 years and were mostly female; a plurality of participants identified as isiXhosa speakers. Most subjects had not completed any secondary schooling. Post-secondary education was rare. Income levels were low among the study participants, with the median income bracket under R5000/year. A substantial majority of participants had been initiated onto ART at least two years prior to the study date (Table 1).

### Adherence levels

More than half of all participants in the quantitative phase (57.3%; *n* = 59/103) indicated some degree of non-adherence to their ARV protocols as determined by their answers to the three adherence questions listed in Table 1. Almost a fifth of participants (18.4%) reported skipping their medication when they felt unwell. Nineteen participants (18.4%) reported not taking their HIV medication the previous weekend. Study participants reported missing a dose of their medications an average of three days per month (median, 2.00 days; standard deviation [s.d.], 4.54), with 25% of all participants reporting missing four or more doses monthly.

#### Levels of stigma among study population

The average total score on the HSS was 30 (median, 30; range, 12–46; s.d., 7.3), reflecting intermediate levels of overall stigma. Almost a fifth of participants (17.5%; *n* = 18/103) reported numerically low stigma scores (≤ 24), indicating high levels of overall stigma. Similar scores were reported across the four subscales (disclosure concerns, negative self-image, concern about public attitudes, personalised stigma). The lowest subscale score (mean, 6.61; s.d., 2.49) was reported for the disclosure concerns category, indicating a higher concern about disclosure of HIV status among study participants as compared to the other types of stigma.

**FIGURE 1:**
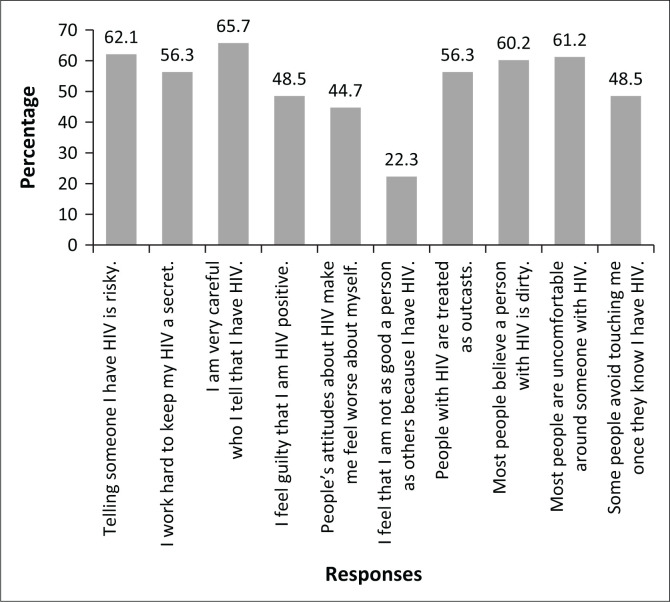
Participant responses to HIV Stigma Scale items. Bars represent the percentage responding ‘strongly agree’ or ‘agree’ to individual items.

As indicated in Figure 1, responses to the individual Likert items within the HSS varied significantly. The percentage of participants who answered ‘strongly agree’ or ‘agree’ to scale items ranged from 65.7% (‘I am very careful who I tell that I have HIV’) to 22.3% (‘I feel that I am not as good a person as others because I have HIV’).

#### Access to needed care

Many subjects indicated major obstacles to receipt of needed care. More than half (52.3%; *n* = 54/103) reported having difficulty getting to the clinic due to issues with transportation, whereas slightly under half of participants (43.7%; *n* = 45/103) reported having some difficulty understanding what their medications did and how often they should take them. Difficulties understanding medical staff were the next most reported issue (38.8%; *n* = 40), followed by lacking familiarity with the clinic (34.0%; *n* = 35/103), not knowing when or how often to take medications (34.0%; *n* = 35/103) and being discouraged from taking medications by family and/or friends (32.0%; *n* = 33/103).

#### Beliefs about antiretrovirals

**FIGURE 2:**
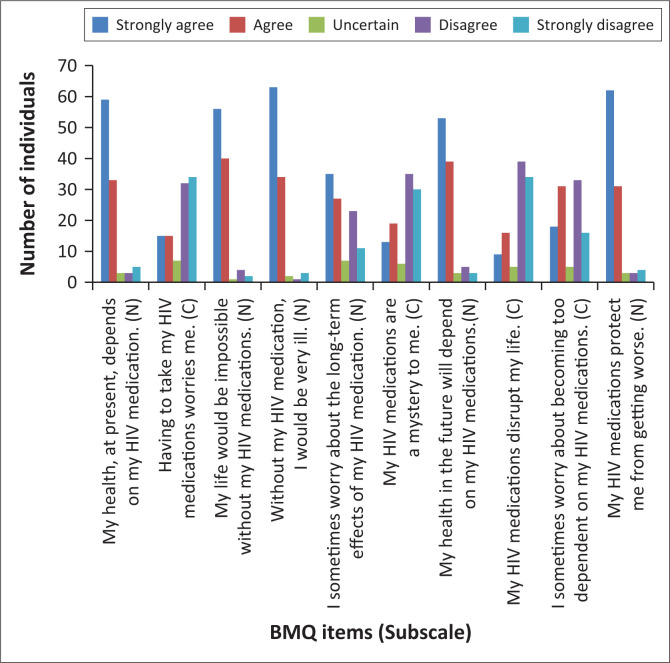
Participant responses to BMQ items (*N* = 103). BMQ items are listed on the horizontal axis, with letters in parentheses indicating the subscale. N, BMQ-Necessity item; C, BMQ-Concerns item; BMQ, Beliefs about Medicine Questionnaire.

Figure 2 shows patients’ responses to the individual BMQ items. Approximately 96.1% of participants scored at or below the BMQ-Necessity scale midpoint (≤ 3 on a scale of 1–5), indicating a high level of belief in the need for their HIV medications (mean, 1.62; median, 1.4; s.d., 0.64). The percentage of patients scoring above the scale midpoint (totally disagree/disagree) ranged from 3.9% (‘Without my HIV medication, I would be very ill’) to 7.8% (‘My health at present depends on my HIV medication; my health in the future will depend on my HIV medications’).

However, over two-fifths of participants (41.7%) scored at or below the BMQ-Concerns scale midpoint, indicating increased concern about their HIV medications (mean, 3.24; median, 3.50; s.d., 1.02). The percentage of patients scoring below the scale midpoint (agree/totally agree) ranged from 24.3% (‘My HIV medications disrupt my life’) to 60.2% (‘I sometimes worry about the long-term effects of my HIV medication’).

#### Knowledge about HIV

Table 2 indicates the percentage of patients who responded correctly to the true/false knowledge items. The responses suggest that the participants have a generally high level of basic knowledge about HIV and their ARVs, with a median score of 3 out of 4 correct. The proportion of participants responding to statements correctly ranged from 58.3% (‘If my HIV medication is causing me to have side effects, it is best to decrease the amount of medication I take that day’; false) to 85.4% (‘ARVs reduce the viral load’; true).

**TABLE 2:** Participant answers to knowledge items.

Statement (answer)	% of participants correct
CD4 counts are used to determine how well the immune system is working. (True)	82.5
If my HIV medication is causing me to have side effects, it is best to decrease the amount of medication I take that day. (False)	58.3
ARVs reduce the viral load. (True)	85.4
If my HIV medication makes me feel better, I can temporarily stop my ARVs. (False)	73.8

ARV, antiretroviral.

#### Predictors of non-adherence

There was a significant inverse correlation between older subject age and the number of days an individual did not take their medication during the previous 30 days (*r* = –0.246, *P* < 0.05). There were no significant correlations between adherence status (adherent vs non-adherent) and other demographic factors, including gender, income level, time on ARVs and education level.

There was a significant relationship between an individual’s negative self-image subscore on the HSS and the number of days that individual did not take their medication in the previous 30 days (Pearson’s *r* = –0.338, *P* < 0.001), indicating that a more negative self-image predicted greater non-adherence. All other associations between stigma scale subscores and adherence were statistically insignificant (*P* > 0.05).

An individual’s score on the BMQ-Necessity was significantly correlated with the number of days that individual skipped their medication during the previous month (*r* = 0.272, *P* < 0.001), reflecting that a lower belief in the necessity of one’s HIV medications predicted a higher number of days being non-adherent to ART. There was a significant correlation between an individual’s score on the BMQ-Concerns and the number of days that individual skipped their medication during the previous month (*r* = –0.266, *P* < 0.001), indicating that greater concern about one’s medication (a lower BMQ-Concerns subscore) was correlated with the degree of non-adherence.

There was a significant difference in the mean knowledge scores between individuals who were classified as adherent (mean, 3.23) versus non-adherent (mean, 2.81) (*P* < 0.05), with non-adherent individuals reporting lower average knowledge about HIV and their ARV protocols.

### Qualitative findings

Several themes emerged from careful review of the transcripts. We describe three major themes selected for their importance in elucidating the reasons behind the differential levels of adherence to HIV medications.

#### ‘They isolate you’ – Fear, stigma and discrimination

In several of the interviews, patients describe a pervasive awareness of the potential for stigmatisation and discrimination because of their HIV status. Several patients opined that an individual’s seropositivity was an important part of one’s social identity in the eyes of community members, and they expanded upon the potential consequences of making one’s positive HIV status public:

‘People … they might just tell somebody [*about your HIV status*]. And they might spread it to everyone else, you know. So that’s how it is. [*They say*], “You know that one is living with HIV, long, long, long.’’ Maybe he’s walking with a girl, maybe he don’t even talk about love. He’s just walking. They say, “Don’t go with that one. Don’t have sex with him because he’s like that.’’’ (Male patient, #38, 48 years old)

‘Maybe, they, like … ignore me. And tell me not to be close to them, to touch anything they use. Stuff like that. … Or [*they*] bad mouth me in front of me. Stuff like that … Yes, I do think that happens sometimes if some other people know.’ (Female patient, #37, 40 years old)

‘You know, sometimes, most of the people … they like to judge people in terms of things about you. “Hey, that one, you must not stay next to that one … because he’s HIV [*positive*]’’ … Even today. They isolate you, most of the things. They don’t want to sit with you. So, you feel ashamed.’ (Male patient, #3, 47 years old)

Patients discussed these instances to contextualise their concerns about inadvertently disclosing their status to the greater community. Several explained that simply being seen taking medications might elicit maltreatment from others, in turn making it difficult to take their ARVs when private space is unavailable:

‘I hide my medications from [*my friends*] … I can see them laughing at other people [*taking their medications*]. So, I don’t want them to laugh at me.’ (Male patient, #26, 33 years old)

‘Yeah, sometimes it’s difficult [*to take your medication*]. Because of people. They are not, how do you say it? They are not safe. Someone will see you doing that. I mean, taking your ARVs. And someone may ask. Or laugh at you. Or you’ll answer, and they will talk to others. … Sometimes you are not treated well in our community. So that is why we are hiding things.’ (Male patient, #38, 48 years old)

#### ‘You’re still drunk or drinking’ – Social environment

Several patients mentioned how some factors in their social environment had either been conducive or detrimental to their health. Social support, including consultations with counsellors as well as forging connections with other people with HIV, emerged as a factor conducive to many patients’ health:

‘The only friends that I can say … that know my status … is the friends who are also HIV [*positive*]. Yes, because we’re like in a club. We know each other. Once that you’re in the club, we know you are HIV [*positive*]. So, I’m not going to say anything around you. Because once you’re there. I know you are HIV [*positive*]. Even you, you won’t say nothing about me. Because you know … once you see me here … You know I’m HIV [*positive*]. So, you know each other, so we don’t talk about that there in the location. Even … when we meet on the road. We just greet each other. There’s nothing else we’re going to talk about. Even if you want to tell them … “Hey, don’t forget your date.” Tomorrow, he’s going to whisper to me … “Hey, tomorrow you’re going to the clinic. Don’t forget’’. Because we know each other.’ (Male patient, #3, 47 years old)

My counselling, when I was first diagnosed I was counselled here, nhe, by [*name withheld for privacy*]. … She was here. She tried to comfort me, telling me everything that this is curable; don’t worry. You’re going to be cured. You’re not going to die; if you keep eating your medication, everything will be fine. People won’t notice anything. She was comforting me … that information made me feel, yes man, I can get through this. So let me get it … so I started using my medication … since then.’ (Male patient, #3, 47 years old)

However, several patients cited alcohol as the most common social deterrent to adherence to HIV medication. The use of alcohol was particularly prevalent in male patients. Some patients mentioned that they feared the adverse effects of mixing alcohol with their medication, so they opted to either drink alcohol or take their medication but not both. Others noted that alcohol use caused them to forget their medications:

‘Yeah, you sometimes, maybe, are drinking too much. And then you go and like just … fell asleep. Maybe wake up at night, which is you can’t take both of your medications.’ (Male patient, #38, 48 years old)

‘Maybe weekends when I drink. Yeah, that weekend when I drink. But I also asked the nurses now recently, “So, what do I do maybe I drink? Do I drink with my medications … with the alcohol?’’ So, the nurse told me it’s fine, I can drink my … it won’t affect me or anything. As long as I take my medication.’ (Male patient, #26, 33 years old)

‘It was … a month. I was stressed. I was busy with alcohol at the time. So, I was always forgetting, those kind of things.’ (Male patient, #147, 54 years old)

#### ‘You can get tired of it’ – HIV and antiretroviral fatigue (‘there’s nothing wrong with me’)

Six of the eight patients interviewed described how taking their ARVs every day often resulted in treatment fatigue. Several of these patients connected this fatigue with their lack of adherence to their HIV medications:

‘Honestly, when you are eating something each and every day, you can get … you can get tired of it. Let’s make … Imagine you make rice and chicken every day. You get sick of eating rice and chicken because you know it’s my everyday food. Yes. So, I can just stop eating rice and chicken, I rather go hungry, yebo? That’s the reason I’m not taking. It’s not because I’m healthy or what …’ (Female patient, #111, 23 years old)

‘Weekends are quite a challenge because sometimes you just feel like not today … I just don’t want to take my medication, and I’ll take it again in the week.’ (Female patient, #52, 40 years old)

Alongside this feeling of being tired of their medications, several patients recounted feeling somewhat justified in skipping doses because of their generally good health. One patient described feeling most frustrated when she felt that her health no longer depended on her ARV regime:

‘Sometimes there is that challenge of why am I taking this and why. … There are those moments. Mostly when I feel … I don’t feel pain or anything. And I feel there’s no use to take this medication. But I have to change that in my mind after that and take it.’ (Female patient, #52, 40 years old)

Some patients mentioned that this feeling of ‘being better’ might explain why many HIV-positive individuals default on their medications:

‘They don’t take it serious. They don’t take it serious. They think once they take their medication, they say, ‘I’m healthy, I see nothing [*wrong*] with me. Why do I have to take this medication? “Ay, let me stop this medication because there is nothing wrong with me. I’m healthy.’’ So, they start from there, not to take their medication.’ (Female patient, #111, 23 years old)

‘Others, they don’t care to take their medication. Sometimes they used to say to me, “If you take and take, you’ll see.’’ They come now better. They stop. If they feel like they need to start again, they run back to the hospital. … Others, they say they’re only tired. Tired to take it every day.’ (Female patient, #80, 41 years old)

## Discussion

More than half of individuals surveyed reported some degree of non-adherence to their ART protocols, with over a quarter having missed four or more doses in the prior 30 days. This figure is significantly higher than several other recent studies investigating suboptimal ART adherence in the greater sub-Saharan African context.^18,19,20^ Younger age was significantly correlated with a lower degree of adherence. The major correlates of low adherence were negative self-image, a lower belief in the necessity of one’s ART regimen and a greater degree of concern about ART. Lower scores on the knowledge section of the questionnaire was also significantly correlated with a lower degree of adherence to ART. The qualitative data in our study suggested that internalised stigma, alcohol use and treatment fatigue also mediated patients’ lack of optimal adherence to ARV medications.

Although the proportion of patients reporting high overall stigma levels was relatively low compared to other studies evaluating HIV-related stigma in similar contexts,^20,21^ several patients indicated that the fear of discrimination related to one’s seropositivity made it difficult to maintain proper adherence in social settings. In particular, all of the men interviewed in the qualitative arm of the study mentioned that taking their medications was especially difficult in social settings, where alcohol use and fear of stigmatisation are prevalent. Although both high degrees of stigma and alcohol use have been previously correlated with low ART adherence,^7,20,22,23,24^ our study suggests that patients are more likely to fear stigmatisation in settings where alcohol is being used. This fear of stigma, combined with concerns about ART–alcohol interactions, resulted in several patients’ decision to not take their ART in social settings, even though patients understood the importance of adherence to their overall health. Moreover, patients who had experienced social isolation or derision because of their status in the past were particularly reluctant to take ART outside their homes, fearing stigmatisation and discrimination by friends, colleagues and community members.

Several patients noted that treatment fatigue made it difficult to take their medications regularly, a finding in line with other recent studies.^25,26,27,28^ In addition, despite an overall high belief in the necessity of their ART and their proficient knowledge about how ART works, many patients felt inclined to skip medications should they experience side effects or feel better. In patients with underlying treatment fatigue, beliefs in the necessity of ART and the inconsequential nature of sometimes skipping medications were often held simultaneously. Thus, an individual’s knowledge of and belief in the positive effects of ART was not sufficient to prevent both fatigue and non-adherence to ART.

The findings of this study suggest ways in which clinicians may improve the identification of patients who may benefit from intensified counselling and/or longitudinal efforts to support ART adherence. An understanding of the major correlates of non-adherence may allow clinicians to better target interventions toward those especially vulnerable to non-optimal adherence levels and address concerns about taking ART. Considering the impact of one’s social environment on adherence, the efficacy of interventions may be enhanced by efforts to help patients navigate such settings.

This study was conducted in a community clinic that serves a low-income patient population in South Africa, with a strong background diversity of study subjects allowing for robust analyses of the correlation between the social drivers of low ART adherence. Two primary limitations constrain the power of inferences one might make based on the findings of this study. Due to the use of convenience sampling and the relatively small sample size, the findings of this study are limited in their generalisability, especially to patient populations with significantly different demographic characteristics. Moreover, social desirability bias might have influenced the manner in which participants answered the surveys or interview questions. Therefore, as several other studies have found, the self-reported non-adherence levels may be an underestimate.^29,30^

## Conclusion

This sequential, exploratory mixed-methods study provides research evidence that suggests that low ART adherence remains highly prevalent in an urban ART clinic in South Africa. We confirmed prior studies showing that negative self-image, lower beliefs in the necessity of one’s medications and higher degrees of concern about one’s treatment regimen were major predictors of low ART adherence. We build upon these data by showing that despite overall high levels of patient belief in the necessity of their medications, lower knowledge with regard to HIV and the requirements of ART also predicted low ART adherence. In addition, our study shows that treatment fatigue and alcohol use pose major challenges for ART adherence, especially among young people who are aware of the necessity of their ART regimens. These data provide improved ways that ART clinics may identify patients at risk for low adherence and suggest interventions that may improve adherence, particularly in at-risk individuals such as younger people, those with low degrees of social support and those engaging in high levels of alcohol use.
